# IMMIGRANT EXPERIENCES AND CARE ARRANGEMENTS LATER IN LIFE: EVIDENCE FROM THE MARIEL BOATLIFT

**DOI:** 10.1093/geroni/igad104.1314

**Published:** 2023-12-21

**Authors:** Freya Diederich

**Affiliations:** University of Bremen, Bremen, Bremen, Germany

## Abstract

The role of public policy in older immigrants’ use of and access to services has mainly been regarded with respect to its legal framework. However, the way public policies are framed and societies’ perceptions of immigrant groups can additionally influence immigrants’ experiences, with long-lasting effects on needs and services use later in life. Still, the importance of these mechanisms has rarely been analyzed. This study uses the Mariel boatlift in 1980 as a case study to assess the relationship between circumstances that surround an immigrants’ cohort and their care arrangement later in life. Cuban immigrants from the Mariel boatlift were perceived in a different way by the federal government and the general population than Cuban immigrants who arrived previously. Based on data from the American Community Survey (2008-2021), care arrangements of older Cubans who arrived in 1980, who are limited in daily activities, and who reside in the Miami area are compared to care arrangements of Cubans that immigrated before. Regression models are estimated and individual characteristics are accounted for. Compared to Cubans who arrived before 1980, Cubans of the Mariel boatlift are more likely to live in the community than in an institutional setting. However, among those living in the community, Cubans of the Mariel boatlift reveal a higher risk of living alone. The results suggest that Cubans of the Mariel boatlift are at higher risk for unmet needs. It is discussed how circumstances that surround immigrants’ cohort of arrival shape immigrants’ services needs and use.

